# Evaluation of the effectiveness of foetal fibronectin as a predictor of preterm birth in symptomatic preterm labour women

**DOI:** 10.1186/s12884-019-2403-7

**Published:** 2019-07-11

**Authors:** Soo Yeun Jun, Ji Young Lee, Hyun-Mi Kim, Mi Ju Kim, Hyun-Hwa Cha, Won Joon Seong

**Affiliations:** Department of Obstetrics and Gynecology, Kyungpook National University Hospital, Kyungpook National University School of Medicine, 807 Hoguk-ro, Buk-gu, Daegu, 702-720 Republic of Korea

**Keywords:** Foetal fibronectin, Cervical length, Preterm birth

## Abstract

**Background:**

The prediction of preterm birth (PTB) is important in the management of symptomatic preterm labour women. We evaluated the effectiveness of the foetal fibronectin (fFN) test for predicting PTB in symptomatic preterm labour women with consideration of physiologic changes in cervical length (CL) during pregnancy.

**Methods:**

This prospective study included 85 women with symptomatic preterm labour of a singleton pregnancy. Positive fFN was defined as a fFN level of > 50 ng/mL in cervicovaginal secretion, while a short CL was defined as that below 25th percentile at the corresponding gestational age. We evaluated effectiveness of the fFN test, CL, and the combination of these two tests, including sensitivity, specificity, positive predictive value (PPV), and negative predictive value (NPV), positive likelihood ratio (LR^+^), negative likelihood ratio (LR^−^) to predict the PTB within 7 and 14 days of testing and PTB at < 34 and 37 weeks of gestation. We also present the odds ratios (ORs) of the test results, defining the women with both negative results as the reference group.

**Results:**

Of the 85 women, 31 (36.5%) showed a positive fFN and 44 (51.8%) had a short CL. PTB occurred within 7 and 14 days of testing and before 34 and 37 weeks of gestation in 17.6, 20.0, 23.5 and 49.4% of the women, respectively. The fFN and CL results showed low predictive effectiveness for the studied outcomes with LR^+^ (fFN, 1.5–1.9; CL, 1.0–1.5) and LR^−^ (fFN, 0.7; CL, 0.7–0.9). The combined use of fFN and CL could not improve these results (LR^+^, 1.4–2.3; LR^−^, 0.7–0.9). However, the risk of PTB before 37 weeks was increased in women with positive fFN but not CL shortening compared to the reference group (odds ratio [OR], 3.8; 95% confidence interval [CI], 1.1–1.3). The risk of PTB before 34 weeks was increased in both positive fFN and CL compared to the reference group (OR, 8.1; 95% CI, 1.9–34.5).

**Conclusion:**

Although, our approach could not improve the ability to predict PTB, it could identify women at risk for delivery before 34 or 37 weeks of gestation. Therefore, it could be used to manage women with symptomatic preterm labour.

## Background

Preterm birth, defined as birth before 37 week of gestation, has a variable incidence of 5–18% and is the major cause of neonatal mortality and morbidity [1, 2]. Preterm infants are vulnerable to neonatal complications including respiratory distress syndrome (RDS), intraventricular haemorrhage (IVH), necrotising enterocolitis (NEC), and retinopathy of prematurity (ROP) [2]. Furthermore, preterm birth is associated with an increased risk of long-term neurodevelopmental impairment and cerebral palsy among survivors [3].

Antenatal corticosteroid (ACS) administration reduces the morbidity and mortality of preterm infants and has been recommended for women at risk of delivery before 34 weeks of gestation. It is known to reduce the infant mortality by 31%, and morbidities by 50% [4], and its administration has been extended to the late preterm period beyond 34 complete weeks of gestation [5]. Although ACS may be effective even if administered only hours before delivery [6], previous reports seem to agree that neonatal benefits are maximised when ACS is administered 24 h to 7 days before delivery [4, 7, 8]. Conversely, ACS administration is known to be associated with adverse neonatal outcomes. In a recent study, a single course of ACS therapy reduced neonatal measurements such as birth weight (− 18%), head circumference (− 9%), body length (− 6%), and placental width (− 6%) compared to those of unexposed infants [9]. Moreover, ACS administration is associated with alterations of the hypothalamus-pituitary-adrenal axis response that persists after birth and could be a possible cause of insulin resistance in adulthood [10]. Therefore, ACS should be administered only to women with a high risk of preterm birth. However, accurately predicting preterm delivery remains difficult.

Healthcare providers have applied several diagnostic tests such as cervical length (CL) and detection of foetal fibronectin (fFN) or phosphorylated insulin-like growth factor-binding protein 1 (phIGFBP-1) in the cervicovaginal fluid for predicting preterm delivery among women in symptomatic preterm labour. Among these tests, CL measurement has been widely used. Although a long CL typically correlates with a lower risk of preterm birth, a short CL does not predict the risk of preterm birth, especially in a low risk group [11].

Fibronectins, a family of ubiquitous proteins found in the plasma and extracellular matrix, are involved in the implantation and maintenance of placental adherence to the maternal decidua [12]. In 1991, Lockwood et al. showed that mechanical or inflammatory-mediated damage to the membranes releases fibronectin into the cervicovaginal fluid [13]; since then, fFN in the cervicovaginal fluid has been used to evaluate the risk of PTB. Many studies have examined the effectiveness of fFN with CL measurement in the prediction of preterm birth, showing varied results [14–16]. However, most studies on the value of CL for predicting PTB did not consider the physiological cervical changes that occur during pregnancy [17] and used a fixed threshold such as 10 mm or 25 mm to define a short CL [14–16, 18, 19].

The objective of this study was to evaluate the effectiveness of fFN in the prediction of preterm birth among women in symptomatic preterm labour while considering the physiologic changes in CL that occur during pregnancy.

## Methods

This prospective observational study was performed at Kyungpook National University Hospital from January 2014 to April 2016. We included women with a singleton pregnancy between 22^0/7^ and 33^6/7^ weeks of gestation with symptoms of preterm labour and intact membranes. Preterm labour was defined as regular uterine contractions occurring at least three times every 10 min irrespective of cervical changes. The exclusion criteria were as follows: confirmed vaginal spotting, history of coitus within 48 h, and suspicion of rupture of membranes, and women under 18 years old. We also excluded intentional preterm delivery of maternal or foetal causes, including preeclampsia, foetal growth restriction, or foetal anomaly.

Each woman who visited our institution with symptomatic preterm labour was first examined using a vaginal speculum to check for vaginal spotting or cervical dilatation. And we obtained three cerviovaginal swab specimens for testing of premature preterm rupture of the membranes (Actim Prom; Medix Biochemica, Finland), bacterial analysis and fFN [20]. When the fFN concentration was > 50 ng/mL, the analyser showed a positive result (fFN enzyme immunoassay and Rapid fFN for the TLi_IQ_ System Hologic) [16]. After taking a cervicovaginal fluid sample, we measured the CL by using transvaginal ultrasonography according to the standard protocol (empty bladder, minimal pressure, and measurement of the shortest length between the internal and external os three times, choosing the shortest of the CL results) [21]. We defined CL, which was measured below the 25th percentile at the corresponding gestational age, as a short CL by using descriptive statistics of CL measurement across gestations [22]. All participating women provided informed consent before participation. This study was approved by the institutional review board (IRB) of Kyungpook National University Hospital, Daegu, Korea (IRB file no. 2018–01-004).

The study outcomes were spontaneous delivery within 7 and 14 days of testing and spontaneous preterm birth before 34 and 37 weeks of gestation. We evaluated the effectiveness (sensitivity, specificity, positive predictive values [PPVs], negative predictive values [NPVs], positive likelihood ratio [LR^+^], negative likelihood ratio [LR^−^]) of the fFN test, CL, and the combination of the two tests for predicting study outcomes. An LR^+^ result > 10 or LR^−^ result < 0.1 are rated to offer strong prediction. Moderate predictive value can be achieved with LRs of 5–10 and 0.1–0.2, whereas those < 5 and > 0.2 are considered minimally predictive [23]. Moreover, we classified the patients into four groups according to their fFN results and CLs: group 1, negative fFN finding and CL above the 25th percentile in the respective gestational age; group 2, positive fFN finding and CL above the 25th percentile in the respective gestational age; group 3, negative fFN finding and CL below the 25th percentile in the respective gestational age; and group 4, positive fFN finding and CL below the 25th percentile in the respective gestational age. Thereafter, the rates of delivery within 7 and 14 days and before 34 and 37 weeks were compared using odds ratios (ORs) obtained from a binary logistic regression analysis with group 1 as the reference group. Statistical analyses were performed using IBM SPSS ver. 19.0 (IBM, Corp, Armonk, NY, USA) and MedCalc version 11.4.1 (MedCalc Software, Mariakerke, Belgium). *P* values < 0.05 were considered statistically significant.

## Results

A total of 151 patients underwent the fFN test due to symptoms of preterm labour in our institution during the study period. Among them, 48 could not be followed up, six had preeclampsia, and 12 had vaginal bleeding. Therefore, 85 patients were included in the study. The general characteristics, pregnancy outcomes, the positive result rate of fFN test and CL are presented in Table 1. At the time of inclusion, the mean gestational age was 29.4 ± 3.6 weeks. Furthermore, 51.8% (44/85) of the study patients showed a CL below the 25th percentile, and 36.5% (31/85) showed a positive finding for fFN. Of the patient cohort, 68.2% (58/85) were administered tocolytics and 65.9% (56/85) were administered ACS during the study period. The mean gestational age at delivery was 35.7 ± 3.7 weeks. 17.6% (15/85) of the patients delivered within 7 days and 20.0% (17/85) of the patients delivered within 14 days. 23.5% (20/85) delivered before 34 weeks of gestation and 49.4% (42/85) of the study patients delivered before 37 weeks of gestation, respectively (Table 1). The detailed clinical information of the 15 cases of delivery within 7 days is presented in Table 2 for exclusion of the possibility of iatrogenic preterm birth. The fFN testing, CL, and combined fFN and CL for predicting study outcomes are presented in Table 3. The combined fFN and CL decreased the sensitivity for predicting study outcomes, while it increased the specificity compared to respective fFN or CL. However, none of these tests showed more than moderate predictive accuracy (LR^+^, 5–10; LR^−^, 0.1–0.2). Table 4 shows the results of the binary logistic test. Group 4 had an increased risk of delivery before 34 weeks (OR, 8.1; 95% CI, 1.9–34.5) compared with group 1. Notably, group 2 had an increased risk of delivery before 37 weeks of gestation (OR, 3.8; 95% CI, 1.1–13.1) compared with group 1.

**Table 1:** Characteristics of the study population

Characteristic	*N* = 85 (%)
Age (years)^a^	31.8 ± 3.9
Maternal BMI (kg/m^2^)^a^	24.55 ± 4.76
Nulliparity (%)	47 (55.2)
Cesarean section (%)	41 (48.2)
Gestational age at inclusion (weeks)^a^	29.4 ± 3.6
Cervical length at inclusion (mm)^b^	22.9 (0–45)
Cervical length < 25th percentile (%)	44 (51.8)
Positive fFN finding (%)	31 (36.5)
Tocolytics use (%)	58 (68.2)
ACS (%)	56 (65.9)
Gestational age at delivery (weeks)^a^	35.7 ± 3.7
Inclusion to delivery interval (days)^b^	45.0 (0–121)
Preterm birth at < 34 weeks (%)	20 (23.5)
Preterm birth at < 37 weeks (%)	42 (49.4)
Delivery within 7 days (%)	15 (17.6)
Delivery within 14 days (%)	17 (20.0)

^a^mean ± SD; ^b^median (range); *BMI* Body mass index, *fFN* foetal fibronectin, *ACS* Antenatal corticosteroid

**Table 2:** Clinical information of 15 women delivering within 7 days

Case	GAD	Mode of delivery	Presentation	Reason for delivery
1	34^2/7^	c/sec	breech	PTL
2	31^1/1^	vaginal	cephalic	PTL
3	36^1/7^	vaginal	cephalic	PTL
4	28^1/7^	vaginal	cephalic	PTL
5	33^4/7^	vaginal	cephalic	PTL
6	28^3/7^	c/sec	cephalic	PTL, non reassuring FHT
7	26^5/7^	c/sec	cephalic	PTL, non reassuring FHT
8	32^2/7^	c/sec	cephalic	PTL, previous c/sec
9	31^3/7^	c/sec	cephalic	PTL, previous c/sec
10	32^4/7^	c/sec	cephalic	PTL, previous c/sec
11	24^4/7^	vaginal	cephalic	PTL
12	35^0/7^	vaginal	cephalic	PTL
13	33^4/7^	c/sec	cephalic	PTL, prevous c/sec
14	31^3/7^	c/sec	cephalic	PTL, previous myomectomy
15	25^3/7^	c/sec	breech	PTL

*GAD* Gestational age at delivery, *PTL* Preterm labour, *FHT*, Fetal hearte tone, *ROM* Rupture of membranes

**Table 3:** Prediction performance of the different diagnostic methods for respective outcomes

	Sensitivity (95% CI)	Specificity (95% CI)	PPV (95% CI)	NPV (95% CI)	LR^+^ (95% CI)	LR^−^
Delivery within 7 days (frequency 15/85), the number of positive results: fFN = 8, CL = 8, combination test = 6						
fFN	(53.3) (30.1–75.3)	67.1 (54.9–77.9)	25.8 (16.3–38.3)	87.0 (79.2–92.2)	1.6 (0.9–2.9)	0.7 (0.4–1.2)
CL	53.3 (26.6–78.7)	48.6 (36.4–60.9)	18.2 (11.6–27.3)	90.2 (72.9–89.8)	1.0 (0.6–1.8)	0.9 (0.5–1.7)
Combination test	40.0 (16.3–67.7	75.7 (63.9–85.2)	26.1 (14.4–42.6)	85.5 (79.2–90.1)	1.6 (0.7–3.5)	0.8 (0.5–1.2)
Delivery within 14 days (frequency 17/85), the number of positive results: fFN = 9, CL = 9, combination test = 6						
fFN	52.9 (27.8–77.0)	67.6 (55.2–78.5)	29.0 (18.9–41.8)	85.2 (77.2–90.7)	1.6 (0.9–2.8)	0.7 (0.4–1.2)
CL	52.9 (27.8–77.0)	48.5 (36.2–61.0)	20.5 (13.4–29.9)	80.5 (70.2–87.8)	1.0 (0.6–1.7)	0.9 (0.1–1.7)
Combination test	35.3 (14.2–61.7)	75.0 (63.0–84.7)	26.1 (14.1–43.1)	82.3(76.1–87.1)	1.4 (0.7–3.0)	0.9 (0.6–1.3)
Delivery at < 34 weeks (frequency 20/85), the number of positive results: fFN = 10, CL = 13, combination test = 9						
fFN	50.0 (27.2–72.8)	67.7 (54.9–78.8)	32.3 (21.4–45.5)	81.5 (73.4–87.6)	1.5 (0.8–2.7)	0.7 (0.5–1.2)
CL	65.0 (40.8–84.6)	52.3 (39.5–64.9)	29.5 (21.7–38.7)	82.9 (71.9–90.2)	1.4 (0.9–2.1)	0.7 (0.3–1.3)
Combination test	45.0 (23.1–68.5)	78.5 (66.5–87.7)	39.1 (24.7–55.7)	82.3 (75.3–87.5)	2.1 (1.1–4.1)	0.7 (0.5–1.1)
Delivery at < 37 weeks (frequency 42/85), the number of positive results: fFN = 20, CL = 26, combination test = 16						
fFN	47.6 (32.0–63.6)	74.4 (58.5–86.5)	64.5 (49.9–76.8)	59.3 (51.0–67.1)	1.9 (1.0–3.4)	0.7 (0.5–0.9)
CL	61.9 (45.6–76.4)	58.1 (42.1–72.9)	59.1 (48.6–68.8)	61.0 (49.6–71.3)	1.5 (1.0–2.3)	0.7 (0.4–1.0)
Combination test	38.1 (23.6–54.4)	83.7 (69.3–93.2)	69.6 (51.2–83.3)	58.1 (51.4–64.5)	2.3 (1.1–5.1)	0.7 (0.5–0.9)

*fFN* foetal fibronectin, *CL* Cervical length, *PPV* Positive predictive value, *NPV* Negative predictive value, *LR* Likelihood ratio

**Table 4:** Comparison of risk of spontaneous preterm delivery within 7 and 14 days after tests and before 34 and 37 weeks of gestation according to fFN and CL results

	Within 7 days	Within 14 days	< 34 weeks	< 37 weeks
Group 1 (*n* = 34) fFN/CL (−/−)				
Group 2 (*n* = 8) fFN/CL (+/−)	2.1 (0.5–8.6)	1.6 (0.4–6.1)	3.8 (1.0–14.7)	3.8 (1.1–13.1)
*P* value	0.30	0.50	0.06	0.04
Group 3 (*n* = 20) fFN/CL (−/+)	3.7 (0.4–36.0)	1.6 (1.0–20.0)	1.7 (0.3–8.4)	1.7 (0.3–8.4)
*P* value	0.25	0.59	0.54	0.51
Group 4 (*n* = 23) fFN/CL (+/+)	4.5 (1.0–20.0)	4.5 (1.0–20.0)	8.1 (1.9–34.5)	2.8 (0.9–8.6)
*P* value	0.05	0.05	0.01	0.08

## Discussion

We observed the effectiveness of fFN testing, CL, and the combination of the two tests with consideration of physiologic cervical changes in the prediction of PTB in symptomatic preterm labour women. None of these tests showed more than moderate accuracy at predicting any of the studied outcomes. However, the combination test could help with the prediction of PTB before 34 weeks of gestation. Interestingly, we found that a positive fFN result without shortening of CL indicated an increased risk of PTB before 37 weeks of gestation.

Since the presence of fFN in the cervicovaginal fluid has been considered a predictor of preterm birth, several studies have evaluated its usefulness. Revah et al. reported that fFN level showed good sensitivity for delivery within 7–10 days of sampling (98% [95% CI, 95–100%]) and delivery before 34 weeks (87% [95% CI, 81–94%]) but showed poor sensitivity for delivery before 37 weeks (54% [95% CI, 51–58%]) in women with symptoms of preterm labour [24]. Furthermore, they reported that fFN testing showed an excellent NPV for the prediction of preterm delivery before 34 and 37 weeks (96% [95% CI, 93–98%] and 85% [95% CI, 84–87%], respectively). Conversely, Greenhagen et al. suggested that a negative fFN finding would correlate with the absence of preterm delivery in low-risk pregnancies; however, it has limited clinical value for predicting preterm birth [25]. Moreover, Berghella et al. reported that fFN testing in singleton gestations with threatened preterm labour is not associated with the prevention of preterm birth or improvement of perinatal outcomes but with higher costs [26]. Compared to previous studies [24, 26], here we demonstrated lower sensitivities, specificities of fFN, and especially NPVs. We assumed that it would be associated with the high rate of follow-up loss (31%, 48/151). Furthermore, 97.9% (47/48) of lost cases showed negative fFN results. Regarding the rate of negative fFN results, we expected that they would deliver at full term at district hospitals. The inclusion of these women could improve our NPV from 87 to 93%. We thought that the high rate of follow-up loss is a feature of the tertiary center like our institution. Therefore, when we use our results in counselling, this high rate of follow up loss should be considered. Meanwhile, several studies have examined the effectiveness of the combination of fFN and CL in the prediction of preterm birth [14, 16, 18]. Schmitz et al. reported that fFN detection in symptomatic women with a short CL yielded excellent NPVs (94 and 99%) for delivery, both before 35 weeks and within 7 days, respectively [14]. Bolt et al. showed that a positive fFN and a short CL (< 25 mm) were associated with a 53% risk of PTB before 37 weeks of gestation compared to a 10% risk in those with both negative results, and they suggested fFN as the first diagnostic tool [16]. Our study group showed higher rate of PTB before 37 weeks of gestation (Group 1 38.2% (13/34) vs. Group 4 69.6% (16/23). It could be related that our study group included women at risk of PTB compared than Bolt’s study. Hincz et al. also suggested that a two-step testing process consisting of fFN as the first step followed by the measurement of CL could be used to predict preterm delivery in women with symptoms of preterm labour [18]. We performed both fFN testing and CL measurements in all our study participants, not a two-step test per se. Our results showed that the combination of fFN and CL measurement could not improve the LR to moderate accuracy (LR^+^, > 5; LR^−^, < 0.2). However, our logistic results showed that the positive findings for both fFN and CL increased the risk of delivery before 34 weeks of gestation. This result could help clinician identify pregnancies that require symptomatic preterm labour management, including ACS administration or the referral to a tertiary centre for preterm birth. This approach is widely used in the management of symptomatic preterm labour women in Korea. Especially when fFN test results are positive, physicians tend to administer ACS or order in utero transfer to tertiary centers. Conversely, when fFN is negative, we can reassure the patient that preterm delivery is not imminent.

Meanwhile, there are emerging evidences that the risk of PTB increases with an increasing fFN concentration [27, 28]. These studies showed various thresholds rather than fixed threshold in qualitative test improve accuracy in prediction of PTB. However, we tried to evaluate the effectiveness of the qualitative fFN with a fixed threshold of 50 ng/ml in which we are actually using in our institution. Therefore we used a fixed qualitative threshold of 50 ng/mL.

It is interesting that group 2 showed an increased risk of delivery before 37 weeks of gestation. Because of high NPV of CL, a long CL defined as at least 25–30 mm is used for reassuring women with preterm labour [11]. However, 50% (4/8) of group 2 in our study delivered before 37 weeks of gestation despite no shortening of CL. Therefore, positive fFN should be a concern even when CL is not short. Recent study also recommends additional fFN testing when CL is between 15 and 30 mm for reducing adverse neonatal outcomes in women with symptoms of preterm labour [19]. We assumed that a positive fFN result indicates damage to the normal foetal membranes, which would be related to eventual preterm birth, even if not immediately. Conversely, group 3 did not show an increased risk of any studied outcomes. This finding was consistent with the known high NPV and low PPV of a short CL in previous studies [29, 30].

Our study has several limitations. Its number of participants was small (*n* = 85) due to the short study period. Furthermore, we could not consider the risk factors of preterm labour in this study. However, the objective of this study was to evaluate the efficacy of qualitative fFN that we currently use at our institution with consideration of CL; therefore, an analysis of the risk factors of preterm birth or quantitative fFN was beyond the study’s scope. Nevertheless, the strength of the study is its prospective observation of patients with preterm labour symptoms who were at a high risk of preterm birth. Our study group showed a similar or higher rate of preterm delivery despite its small sample size [16, 27]. This finding implies that our study result could be applicable to women at increased risk of PTB. Furthermore, we considered the physiological cervical changes that occur in the third trimester [11, 17], unlike other studies of CL for predicting PTB [14–16, 18, 19]. We also presented ORs for respective study outcomes, defining the women with both negative results as the reference group, making our results easily applicable for counselling symptomatic preterm labour women. And finally, this is the first study in South Korea to evaluate the preterm birth rate using the combination of fFN and CL.

## Conclusions

In conclusion, we found that the combination of fFN and CL with consideration of the physiologic changes in CL could not improve PTB prediction accuracy. However, it could help clinicians identify the women at risk of delivery before 34 weeks or 37 weeks. Therefore, it could be used in the management of women with symptomatic preterm labour.

## Data Availability

The datasets acquired and/or analysed during the current study are available from the corresponding author on reasonable request.

## Abbreviations

CL: Cervical length
fFN: Foetal fibronectin
IVH: Intraventricular haemorrhage
LR^−^: Negative likelihood ratio
LR^+^: Positive likelihood ratio
NPV: Negative predictive value
PPV: Positive predictive value
PTB: Preterm birth
RDS: Respiratory distress syndrome

## References

[1] Blencowe H, Cousens S, Oestergaard MZ, Chou D, Moller AB, Narwai R (2012). National, regional, and worldwide estimates of preterm birth rates in the year 2010 with time trends since 1990 for selected countries: a systematic analysis and implications. Lancet.

[2] Liu L, Oza S, Hogan D, Chu Y, Perin J, Zhu J (2016). Global, regional, and national causes of under-5 mortality in 2000-15: an updated systematic analysis with implications for the sustainable development goals. Lancet.

[3] Blencowe H, Lee AC, Cousens S, Bahlim A, Narwal R, Zhong N (2013). Preterm birth-associated neurodevelopmental impairment estimates at regional and global levels for 2010. Pediatr Res.

[4] Roberts D, Dalziel S. Antenatal corticosteroids for accelerating fetal lung maturation for women at risk of preterm birth. Cochrane Database Syst Rev. 2006;19:Cd004454. 10.1002/14651858.CD004454.pub2 16856047

[5] Committee on Obstetric Practice (2017). Committee opinion No 713: Antenatal Corticosteroid Therapy for Fetal Maturation. Obstet Gynecol.

[6] Norman M, Piedvache A, Borch K, Huusom LD, Bonamy AE, Howell EA (2017). Association of Short Antenatal Corticosteroid Administration-to-Birth Intervals with Survival and Morbidity among Very Preterm Infants: results from the EPICE cohort. JAMA Pediatr.

[7] Melamed N, Shah J, Sorisham A, Yoon EW, Lee SK, Shah PS (2015). Association between antenatal corticosteroid administration-to-birth interval and outcomes of preterm neonates. Obstet Gynecol.

[8] Kuk JY, An JJ, Cha HH, Choi SJm Vargas JE, Oh SY (2013). Optimal time interval between a single course of antenatal corticosteroids and delivery for reduction of respiratory distress syndrome in preterm twins. Am J Obstet Gynecol.

[9] Audette MC, Challis JR, Jones RL, Sibley CP, Matthews SG (2014). Synthetic glucocorticoid reduces human placental system a transport in women treated with antenatal therapy. J Clin Endocrinol Metab.

[10] Schmitz T (2016). Prevention of preterm birth complications by antenatal corticosteroid administration. J Gynecol Obstet Biol Reprod (Paris).

[11] Lim K, Butt K, Crane JM (2011). Diagnostic imaging committee; family physicians advisory committee; maternal fetal medicine committee. SOGC clinical practice guideline. Ultrasonographic cervical length assessment in predicting preterm birth in singleton pregnancies. J Obstet Gynaecol Can.

[12] Leeson SC, Maresh MJ, Martindale EZ, Mahmood T, Muotune A, Hawkes N (1996). Detection of fetal fibronectin as a predictor of preterm delivery in high risk asymptomatic pregnancies. Br J Obstet Gynaecol.

[13] Lockwood CJ, Senyei AE, Dische MR, Casal D, Shah KD, Thung SN (1991). Fetal fibronectin in cervical and vaginal secretions as a predictor of preterm delivery. N Engl J Med.

[14] Schmitz T, Maillard F, Bessard-Bacquaert S, Kayem G, Fulla Y, Cabrol D (2006). Selective use of fetal fibronectin detection after cervical length measurement to predict spontaneous preterm delivery in women with preterm labor. Am J Obstet Gynecol.

[15] Smith V, Devance D, Begley CM, Clarke M, Higgins S (2007). A systematic review and quality assessment of systematic reviews of fetal fibronectin and transvaginal length for predicting preterm birth. Eur J Obstet Gynecol Reprod Biol.

[16] Bolt LA, Chandiramani M, DeGreeff A, Seed PT, Kurtzman J, Shennan AH (2011). The value of combined cervical length measurement and fetal fibronectin testing to predict spontaneous preterm birth in asymptomatic high-risk women. J Matern Fetal Neonatal Med.

[17] Liabsuetrakul T, Suntharasaj T, Suwanrath C, Leetanaporn R, Rattanaprueksachart R, Tuntiseranee P (2002). Serial translabial sonographic measurement of cervical dimensions between 24 and 34 weeks’ gestation in pregnant Thai women. Ultrasound Obstet Gynecol.

[18] Hincz P, Wilczynski J, Kozarzewski M, Szaflik K (2002). Two-step test: the combined use of fetal fibronectin and sonographic examination of the uterine cervix for prediction of preterm delivery in symptomatic patients. Acta Obstet Gynecol Scand.

[19] van Baaren GJ, Vis JY, Wilms FF, Qudijk MA, Kwee A, Porath MM (2018). Cost-effectiveness of diagnostic testing strategies including cervical-length measurement and fibronectin testing in women with symptoms of preterm labor. Ultrasound Obstet Gynecol.

[20] Fuchs F, Houllier M, Leparco S, Guyot A, Senat MV, Fernandez H (2017). Performanc of cervical phIGFBP-1 test alone or combined with shoret cervical length to predict spontaneous preterm birth in symptomatic women. Sci Rep.

[21] Berghella V, Bega G, Tolsa JE, Berghella M (2003). Ultrasound assessment of the cervix. Clin Obstet Gynecol.

[22] Salomon LJ, Diaz-Garcia C, Bernard JP, Ville Y (2009). Reference range for cervical length throughout pregnancy: non-parametric LMS-based model applied to a large sample. Ultrasound Obstet Gynecol.

[23] Jaeschke R, Guyatt GH, Sackett DL (1994). Users’ guides to the medical literature. III. How to use an article about a diagnostic test. B. What are the results and will they help me in caring for my patients? The Evidence-Based Medicine Working Group. JAMA.

[24] Revah A, Hannah ME, Sue-A-Quan AK (1998). Fetal fibronectin as a predictor of preterm birth: an overview. Am J Perinatol.

[25] Greenhagen JB, Van Wagoner J, Dudley D, Hunter C, Mitchell M, Logsdon V (1996). Value of fetal fibronectin as a predictor of preterm delivery for a low-risk population. Am J Obstet Gynecol.

[26] Berghella V, Saccone G (2016). Fetal fibronectin testing for prevention of preterm birth in singleton pregnancies with threatened preterm labor: a systematic review and metaanalysis of randomized controlled trials. Am J Obstet Gynecol.

[27] Hermans FJR, Bruijn MMC, Vis JY, Wilms FF, Oudijk MA, Porath MM (2015). Risk stratification with cervical length and fetal fibronectin in women with threatened preterm labor before 34 weeks and not delivering within 7 days. Acta Obstet Gynecol Scand.

[28] Bruijn M, Vis JY, Wilms FF, Oudijk MA, Kwee A, Porath MM (2016). Quantitative fetal fibronectin testing in combination with cervical length measurement in the prediction of spontaneous preterm delivery in symptomatic women. BJOG..

[29] Ness A, Visntine J, Ricci E, Berghella V (2007). Does knowledge of cervical length and fetal fibronectin affect management of women with threatened preterm labor? A randomized trial. Am J Obstet Gynecol.

[30] Peixoto AB, da Cunha Caldas TMR, Tahan LA, Petrini CG, Martins WP, Costa FDS (2017). Second trimester cervical length measurement for prediction spontaneous preterm birth in an unselected risk population. Obstet Gynecol Sci.

